# Dual-specificity phosphatases 22-deficient T cells contribute to the pathogenesis of ankylosing spondylitis

**DOI:** 10.1186/s12916-023-02745-6

**Published:** 2023-02-10

**Authors:** Ming-Han Chen, Huai-Chia Chuang, Yi-Chen Yeh, Chung-Tei Chou, Tse-Hua Tan

**Affiliations:** 1 Division of Allergy, Immunology & Rheumatology, Taipei Veterans General Hospital, Taipei, Taiwan; 2 Faculty of Medicine, National Yang Ming Chiao Tung University, Taipei, Taiwan; 3 Immunology Research Center, National Health Research Institutes, Zhunan, Taiwan; 4 Department of Pathology and Laboratory Medicine, Taipei Veterans General Hospital, Taipei, Taiwan; 5 Department of Pathology & Immunology, Baylor College of Medicine, Houston, Texas USA

**Keywords:** DUSP22, Ankylosing spondylitis, Tumor necrosis factor-α, Interferon-γ, Interleukin-17A

## Abstract

**Background:**

Dual-specificity phosphatases (DUSPs) can dephosphorylate both tyrosine and serine/threonine residues of their substrates and regulate T cell-mediated immunity and autoimmunity. The aim of this study was to investigate the potential roles of DUSPs in ankylosing spondylitis (AS).

**Methods:**

Sixty AS patients and 45 healthy controls were enrolled in this study. Associations of gene expression of 23 DUSPs in peripheral T cells with inflammatory cytokine gene expression and disease activity of AS were analyzed. Finally, we investigated whether the characteristics of AS are developed in DUSP-knockout mice.

**Results:**

The mRNA levels of DUSP4, DUSP5, DUSP6, DUSP7, and DUSP14 in peripheral T cells were significantly higher in AS group than those of healthy controls (all *p* < 0.05), while DUSP22 (also named JKAP) mRNA levels were significantly lower in AS group than healthy controls (*p* < 0.001). The mRNA levels of DUSP4, DUSP5, DUSP6, DUSP7, and DUSP14 in T cells were positively correlated with mRNA levels of tumor necrosis factor-α (TNF-α), whereas DUSP22 was inversely correlated (all *p* < 0.05). In addition, inverse correlations of DUSP22 gene expression in peripheral T cells with C-reactive protein, erythrocyte sedimentation rate, and Bath Ankylosing Spondylitis Disease Activity Index (BASDAI) were observed (all *p* < 0.05). More importantly, aged DUSP22 knockout mice spontaneously developed syndesmophyte formation, which was accompanied by an increase of TNF-α^+^, interleukin-17A^+^, and interferon-γ^+^ CD3^+^ T cells.

**Conclusions:**

DUSP22 may play a crucial role in the pathogenesis and regulation of disease activity of AS.

**Supplementary Information:**

The online version contains supplementary material available at 10.1186/s12916-023-02745-6.

## Background

Dual-specificity phosphatases (DUSPs), currently including 25 members, can dephosphorylate both tyrosine and serine/threonine residues of their substrates [1]. DUSPs are able to regulate the activities of MAP kinases [1–3], which play central roles of thymocyte development, T helper (Th) cell polarization, and T cell signaling [4, 5]. DUSPs also can dephosphorylate non-MAP kinase proteins, such as TAK1, occludin, and focal adhesion kinase (FAK) [1, 6–8]. DUSP4 negatively regulates CD4^+^ T cell proliferation through dephosphorylating STAT5 [1]. DUSP22, also named JNK pathway-associated phosphatase (JKAP), is a JNK activator [1, 9] and FAK inactivator [10]. DUSP22 directly inactivates the tyrosine kinase Lck by dephosphorylating tyrosine-394 residue during T cell receptor signaling [11]. Furthermore, DUSP22 dysregulation is associated with human lupus nephritis [12].

Ankylosing spondylitis (AS) belongs to seronegative spondyloarthropathies (SpA), which is characterized by sacroiliitis and inflammatory back pain with stiffness [13, 14]. Syndesmophyte formation may develop if disease progresses, resulting in working disability and health-related quality-of-life impairment. The pathogenesis of AS is unclear; current evidences indicate that human leukocyte antigen (HLA)-B27 plays a critical pathogenic role through its autoantigen- or misfolding-induced unfolded protein response, leading to the induction of proinflammatory cytokines and chemokines [15]. In addition, T cell activation may also contribute to the pathogenesis of AS [16, 17]. An induction of T helper type 1 (Th1) cell-induced proinflammatory cytokines and a reduction of Th2 cell-induced anti-inflammatory cytokines occur in AS patients with high active disease activity [18]. Tumor necrosis factor-α (TNF-α), a proinflammatory cytokine mainly secreted by NK cells, T cells, monocytes, and macrophages, plays a pathogenic role in AS [19]. TNF-α mediates tissue inflammation, bone erosion, and new bone formation by stimulation of TNF receptor 1 [20]. Th17 cytokines also contribute to enthesitis, osteitis, bone erosion, and new bone formation, which are the hallmark skeletal features associated with the pathophysiology of axial SpA [21]. Mechanistically, dendritic cell-secreted interleukin (IL)-12 and IL-23 activate Th17, Th22, and Th1 cells, leading to the production of the inflammatory cytokines IL-17, interferon-γ (IFN-γ), TNF-α, and IL-22. Indeed, clinical trials using TNF-α blockade or IL-17A blockade show some beneficial effects for AS patients [22–24].

DUSP-family phosphatases regulate T cell-mediated immune responses and the production of proinflammatory cytokines, which play crucial roles in the pathogenesis of inflammatory arthritis [4, 5, 11, 25]. DUSP22 levels in synovium and serum are inversely correlated with inflammation and disease activity in patients with rheumatoid arthritis (RA) [26]. Moreover, downregulation of DUSP22 protein in T cells is a prognostic biomarker for SLE nephritis [12]. To date, the importance of DUSPs in AS remains unknown, and this study aimed at investigating the potential roles of DUSPs in AS. We explored whether gene expression levels of various DUSPs in T cells could be potential diagnostic biomarkers for AS. Associations of DUSP levels in T cells with inflammatory markers and disease activity of AS were analyzed. Correlations of gene expression levels between individual DUSPs and inflammatory cytokines were also interrogated. Finally, we investigated whether the characteristics of AS manifested in DUSP-knockout (KO) mice.

## Methods

### Patients

The present study enrolled patients with AS and healthy controls (HCs) at Taipei Veterans General Hospital. Diagnosis of AS was based on 1984 modified New York AS criteria [27]. Clinical, laboratory, and radiographic findings were recorded. C-reactive protein (CRP) and erythrocyte sedimentation rate (ESR) were used as markers for inflammation, while Bath Ankylosing Spondylitis Disease Activity Index (BASDAI), Functional Index (BASFI), and Patient Global Score (BAS-G) were used to evaluate disease activity and function [28–30]. The modified Stokes Ankylosing Spondylitis Spinal Score (mSASSS) was used to assess radiological progression of AS [31]. The study has been approved by the Institutional Review Board of Taipei Veterans General Hospital (2015-12-016AC). Informed consent forms were obtained from all patients participating in this study. We used G*Power to determine the minimum effect size. The effect size value was based on a previous publication [12]. The minimum sample size was 68 when the level of power was 0.80, while the minimum sample size was 88 when the level of power was 0.90. In this study, 105 individuals (60 AS patients and 45 healthy controls) were enrolled.

### Determination of gene expression of DUSPs and inflammatory cytokines in peripheral blood T cells

Peripheral blood was obtained from enrolled subjects. After lysis of red blood cells by ammonium-chloride-potassium (ACK) buffer, peripheral leukocytes (PBLs) were isolated by centrifugation. T cells were then purified from PBLs of study subjects by negative selection for CD14, CD19, CD235A, and CD11b using immunomagnetic beads (BD Biosciences, San Jose, CA, USA). The purity of the isolated human T cells is more than 90% (Fig. S1). The mRNA levels of DUSPs and inflammatory cytokines, including IFN-γ, TNF-α, IL-6, and IL-17A, were determined by quantitative real-time polymerase chain reaction (qRT-PCR). The primers used for detection of DUSPs are shown in supplementary Table S1. Total RNAs were extracted from purified T cells using the 6100 Nucleic Acid PrepStation (Biosystems, Foster City, CA, USA). Then, 500 ng of total RNAs were converted to cDNA using SuperScript™ III First-Strand Synthesis System (Thermo Fisher Scientific, Waltham, MA, USA). The cDNAs (200 ng) were reversely transcribed and amplified by PCR with Maxima SYBR Green qPCR Master Mix (2X) (Thermo Fisher Scientific, Waltham, MA, USA) using the StepOne Plus™ Real-Time PCR System (Thermo Fisher Scientific). Each reaction was performed in duplicate. The expression levels of DUSPs were normalized to glyceraldehyde 3-phosphate dehydrogenase (GAPDH), which was used as a control.

### Mouse model

The DUSP22 knockout (KO) mouse line in C57BL/6J background was generated by the CRISPR/Cas9 approach. DUSP22 KO mice were backcrossed to C57BL/6J background for 10 generations. PCR analysis was used to determine the genotype of the offspring. Mice were randomly selected for in vivo animal experiments. To characterize T cell development of DUSP22 KO and wild-type mice, 5-week-old mice were sacrificed by CO_2_. The thymocytes of the sacrificed mice were subjected to flow cytometry analysis using antibodies against individual surface markers [11]. To determine whether DUSP22 deficiency influences the development of syndesmophyte, the spine image of 1-year-old DUSP22 KO and wild-type mice were determined by micro-computed tomography (μCT). The mice were anaesthetized using isoflurane during the period of image capture. After being sacrificed by CO_2_, paraffin-embedded spine sections of 1-year-old DUSP22 KO and wild-type mice were subjected to histology and immunohistochemistry. All mouse studies were carried out under the approval of the Health National Research Institutes Animal Ethics Committee.

### Immunocytochemistry and immunofluorescence assays

Skeletons were collected from mice and fixed in 10% neutral-buffered formalin for 4 days at 4°C, followed by decalcification with 14% ethylenediaminetetraacetic acid (EDTA) for 2 weeks. Tissue sections were stained with hematoxylin and eosin (H&E) according to standard protocols. Anti-osteocalcin monoclonal antibody (clone #E-6) was purchased from Santa Cruz Biotechnology (Dallas, TX, USA). Safranin O (#S2255) and fast green FCF (#F7262) were purchased from Sigma-Aldrich (Darmstadt, Germany). For immunofluorescence experiments, preparations were incubated for 1 h at room temperature with Alexa Fluor® 488- or PE-conjugated anti-mouse CD3 antibody (clone #17A2, BioLegend, San Diego, CA, USA), APC-conjugated anti-TNF-α antibody (clone #MP6-XT22, BioLegend), FITC-conjugated anti-IL-17A antibody (clone #REA660, Miltenyi Biotec, Bergisch Gladbach, Germany), and FITC-conjugated anti-IFN-γ antibody (clone #XMG1.2, BioLegend). Homemade anti-DUSP22 monoclonal antibody (clone #C8), recognizing both human and mouse DUSP22 proteins, was generated by immunization of mice with peptides (murine DUSP22 epitope: ^170^GKYKEQGRTEPQPGARRWSS^189^). 4′,6-diamidino-2-phenylindole (DAPI) was used for counterstaining (Merck, Darmstadt, Germany). Fluorescence was detected using a fluorescence microscope (Leica TCS SP5II).

### Flow cytometry analysis

For human peripheral blood leukocyte (PBL) analyses, PBLs were immediately treated with Golgi-stop without any stimulation and then stained with anti-human CD3-PE-Cy7 (clone #SK7, BD Biosciences) and anti-human CD4-pacific blue (clone #RPA-T4, BD Biosciences) antibodies for 3 h. For intracellular cytokines/DUSP22 staining, the surface marker-labelled cells were permeabilized by Cytofix/Cytoperm buffer (BD Biosciences) overnight. After washing with Perm-Wash buffer, the cells were incubated with anti-human IL-17A-Alex647 (clone #N49-653, BD Biosciences, 1:50 dilution), anti-IFN-γ-FITC (clone #XMG1.2, BioLegend, 1:50 dilution), anti-human TNF-α antibody (clone #MP6-XT22, BioLegend, 1:50 dilution), or anti-DUSP22 (clone #C8, 1:50 dilution) antibody for 3 h.

### Statistical analysis

We used the ARRIVE guideline when writing this paper. We used G*Power to determine the minimum effect size. The baseline characteristics were analyzed using the Student’s *t* tests for continuous variables. The chi-squared test or Fisher’s exact test was used to compare the baseline characteristics between AS and HC groups. Mean mRNA levels of DUSPs between AS and HC groups were compared using the Mann-Whitney *U* test. Spearman correlations were used to assess the associations of DUSP mRNA levels with disease activity and laboratory results. Receiver operator characteristics (ROC) analysis was conducted, and area under the curve (AUC) was calculated to measure the prediction accuracy. All the *p* values shown are 2-tailed and were regarded as significant when less than 0.05. Data were analyzed with SPSS software, version 26.0 (IBM SPSS Statistics for Windows, IBM, Armonk, New York, USA).

## Results

### Demographic findings

The demographic and clinical characteristics of the participants are summarized in Table 1. Average ages of 60 AS and 45 HCs were 37.9 and 39.3 years, respectively. Forty-two (70.0%) AS patients and 34 (75.6%) HCs were men. The age and gender ratios were not significantly different between two groups. The median of disease duration from AS diagnosis was 8.0 years, and 58 (96.7%) AS patients were positive for the HLA-B27 antigen. Laboratory assessments showed that the mean levels of ESR was 23.3 mm/h, while the median level of CRP was 0.9 mg/dl (mean = 1.3 mg/dl) in AS group. Notably, half of the AS patients had CRP levels ≥ 1.0 mg/dl, which is associated with high disease activity and a predictor of poor TNF-α inhibitor retention rate [32]. All AS patients had sacroiliitis with mean grade of 2.7, while mean mSASSS score was 9.5. The average scores of BASDAI, BASFI, and BAS-G were 4.5, 2.4, and 4.5, respectively. Biologics were prescribed for 11 (18.3%) AS patients who were refractory to nonsteroidal anti-inflammatory drug treatment.

**Table 1:** Demographic characteristics of enrolled patients with AS and healthy controls

	AS ***N*** = 60	Healthy controls ***N*** = 45	***p*** value
Age, years	37.9 ± 13.5	39.3 ± 13.2	0.511
Gender, male	42 (70.0)	34 (75.6)	0.527
Disease duration, years	8 (3, 17)	-	-
HLA-B27 positivity	58 (96.7)	-	-
mSASSS (0–72)	2 (0, 9)	-	-
Sacroiliitis grade (0–4)	3 (2, 3)	-	-
ESR, mm/h	23.3 ± 17.9	-	-
CRP, mg/dl	0.9 (0.2, 2.1)	-	-
BASDAI (0–10)	4.5 ± 2.2	-	-
BASFI score (0–10)	2.4 ± 2.4	-	-
BAS-G score (0–10)	4.5 ± 2.5	-	-
Biologic used	11 (18.3%)	-	-

*Abbreviation*: *BASDAI* Bath Ankylosing Spondylitis Disease Activity Index, *BASFI* BAS Functional Index, *BAS-G* BAS Patient Global Score, *CRP* C-reactive protein, *ESR* Erythrocyte sedimentation rate, *HLA* Human leukocyte antigen, *mSASSS* Modified Stokes Ankylosing Spondylitis Spinal Score

Data are presented as frequency (percentage), mean ± standard deviation, or median (interquartile range)

### Comparison of DUSP mRNA levels in peripheral T cells between AS patients and HCs

**Fig. 1:**
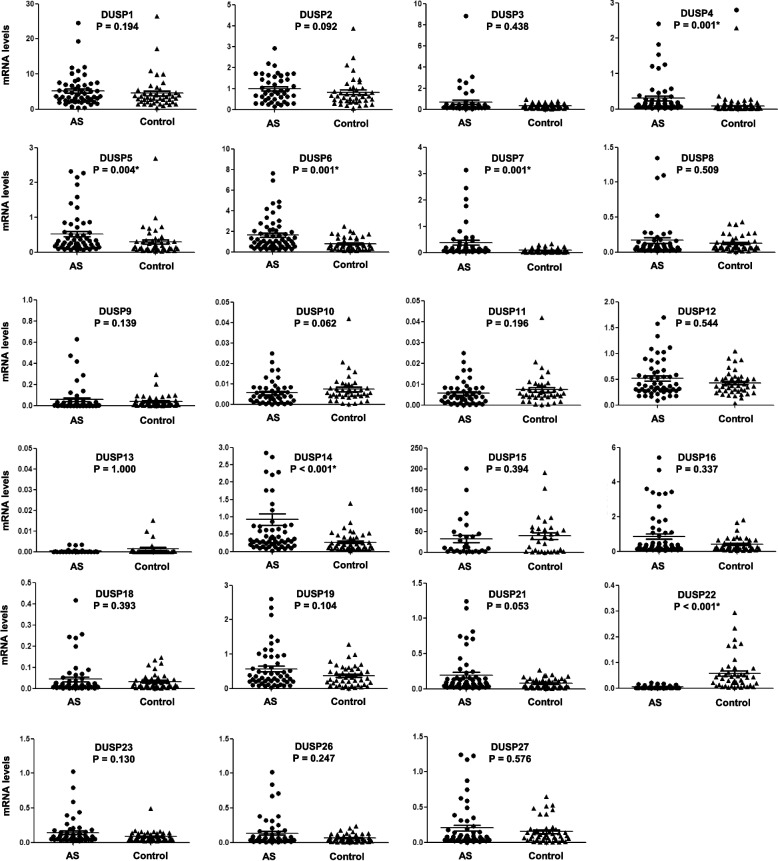
The mRNA expression levels of DUSP-family phosphatases in peripheral blood T cells of patients with AS and age- and sex-matched healthy controls. Blood samples were obtained from 60 AS patients, as well as 45 age- and sex-matched healthy controls. The mRNA levels of 23 DUSPs were measured by quantitative real-time polymerase chain reaction. One representative result of two experiments is shown. * *p* < 0.05. The mRNA levels of individual DUSPs were normalized to that of GAPDH

**Fig. 2:**
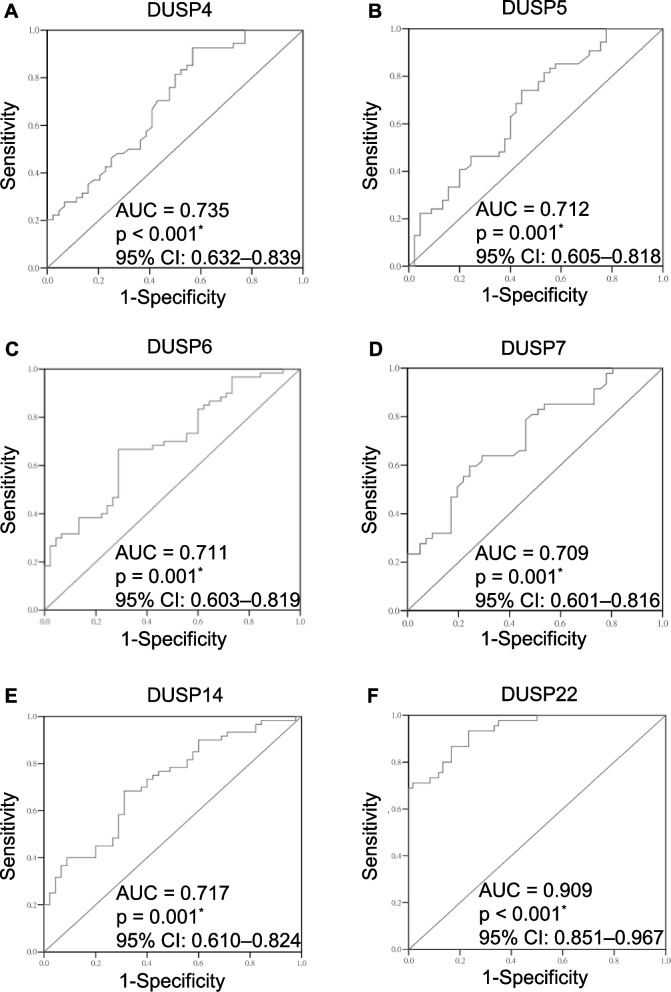
Receiver operating characteristic curve analysis of DUSP mRNA level in peripheral blood T cells of patients with AS. Area under the receiver operating characteristic curve (AUC) analysis showing diagnostic accuracy of DUSP4, DUSP5, DUSP6, DUSP7, DUSP14, and DUSP22 mRNA levels in peripheral T cells for AS (**A–F**). * *p* < 0.05

We studied DUSP gene expression levels in T cells of enrolled subjects. As shown in Fig. 1, we found that the mRNA levels of DUSP 4, DUSP5, DUSP6, DUSP7, and DUSP14 in peripheral T cells were significantly higher in AS group than those in HCs (all *p* < 0.005), whereas DUSP22 mRNA levels were significantly lower in AS group (*p* < 0.001). No significant difference of DUSP mRNA levels was observed between AS patients with and without biologic treatment (all *p* > 0.05). To determine whether the mRNA levels of the abovementioned DUSPs in T cells were useful diagnostic biomarkers for AS, the diagnostic utility of DUSP gene expression levels were analyzed by ROC analysis. As shown in Fig. 2, the mRNA levels of DUSP4, DUSP5, DUSP6, DUSP7, DUSP14, and DUSP22 in T cells were able to accurately detect AS patients (all *p* < 0.05). Among the 6 candidate biomarkers of DUSPs, the mRNA levels of DUSP22 in T cells showed the highest AUC (AUC = 0.9047, 95% confidence interval [CI] 0.851 to 0.967, *p* < 0.001), and the best cut-off value of 0.019 was determined (sensitivity 68.1%, specificity 98.4%).

### Correlations of the gene expression of DUSPs and inflammatory cytokines in peripheral T cells

We next analyzed the correlations of gene expression of these 6 candidate DUSPs and inflammatory cytokines, including TNF-α, IFN-γ, IL-6, and IL-17A, in human peripheral blood T cells obtained from 25 enrolled subjects. As shown in Table 2 and Fig. S2, the mRNA levels of 5 DUSPs (DUSP4, DUSP5, DUSP6, DUSP7, and DUSP14) in T cells were positively associated with TNF-α mRNA level (all *p* < 0.001), whereas the mRNA levels of DUSP22 was negatively correlated with TNF-α mRNA levels (*r* = −0.644, *p* < 0.001). The mRNA levels of all 6 candidate DUSPs did not show any significant correlations with the mRNA levels of IFN-γ and IL-6 in peripheral T cells (all *p* > 0.05). IL-17A mRNA in peripheral blood T cells was undetectable in 9 of 25 subjects; thus, the correlation between IL-17A mRNA levels with those of DUSPs could not be evaluated. As an alternative approach, we examined the protein levels of cytokines (particular IL-17A and IFN-γ) by flow cytometry analysis using peripheral blood samples from the second batch of AS patients and healthy controls (Table S2). Unlike cytokine mRNA levels, we found that the frequencies of IL-17A-producing T cells and IFN-γ-producing T cells were increased in peripheral blood leukocytes (PBLs) of AS patients compared to those of healthy controls (Fig. S3). The frequency of TNF-α-producing T cells was also increased in PBLs of AS patients (Fig. S3). Downregulation of DUSP22 mRNA levels is most likely to be a biomarker for AS patients; therefore, we further examined the DUSP22 protein levels in T cells using flow cytometry. Consistent with mRNA levels, the frequency of DUSP22-positive T cells was decreased in AS patients compared to that of healthy controls (Fig. S3). These results showed that DUSP22 downregulation and IL-17A/TNF-α/IFN-γ induction occurred in T cells of AS patients.

**Table 2:** Correlations between the mRNA levels of DUSPs and proinflammatory cytokines in peripheral T cells in patients with ankylosing spondylitis

	DUSP4		DUSP5		DUSP6		DUSP7		DUSP14		DUSP22	
	*r*	*p* value	*r*	*p* value	*r*	*p* value	*r*	*p* value	*r*	*p* value	*r*	*p* value
TNF-α	0.889	< 0.001^*^	0.826	< 0.001^*^	0.724	< 0.001^*^	0.895	< 0.001^*^	0.791	< 0.001^*^	−0.644	< 0.001^*^
IFN-γ	−0.325	0.188	−0.135	0.593	−0.088	0.721	−0.315	0.203	−0.182	0.455	−0.044	0.858
IL-6	−0.071	0.879	−0.429	0.337	0.048	0.911	−0.107	0.819	0.000	1.000	−0.190	0.651

*Abbreviation*: *IFN-γ* Interferon-γ, *IL-6* Interleukin-6, *TNF-α* Tumor necrosis factor-α

*Significant *p* value (< 0.05)

### Relationship between DUSP mRNA levels and the disease activity of patients with AS

**Fig. 3:**
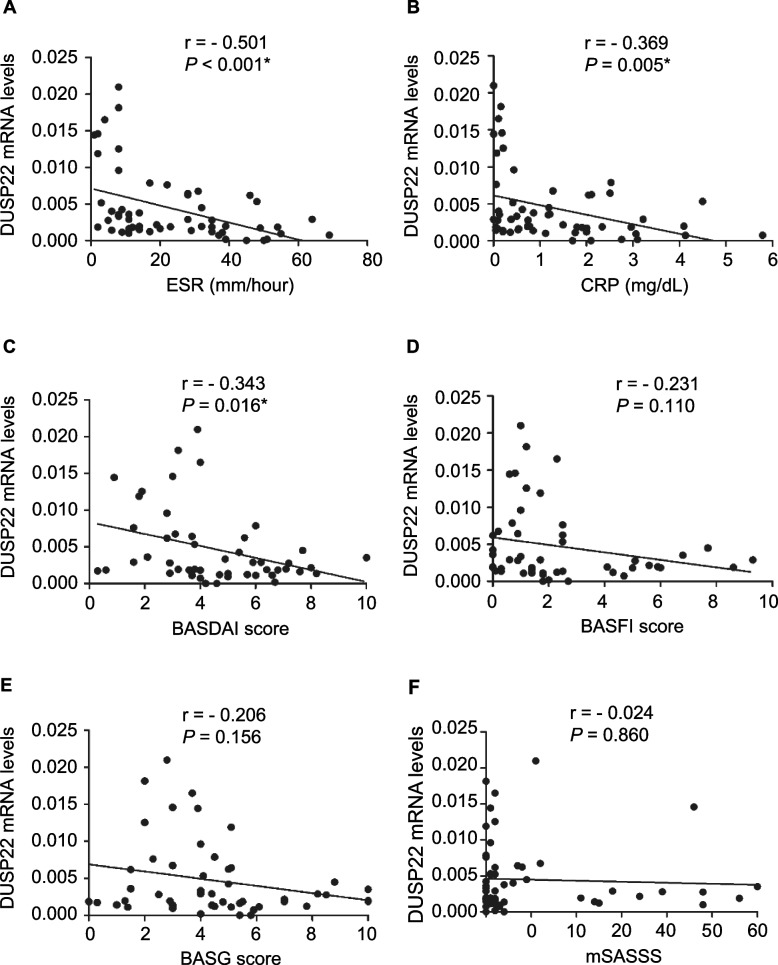
DUSP22 gene expression is associated with disease activity of AS. Association between DUSP22 mRNA levels in peripheral blood T cells and erythrocyte sedimentation rate (ESR) (**A**), C-reactive protein (CRP) levels (**B**), Bath Ankylosing Spondylitis Disease Activity Index (BASDAI) (**C**), BAS Functional Index (BASFI) (**D**), and BAS Patient Global Score (BAS-G) (**E**), and the modified Stoke Ankylosing Spondylitis Spinal Score (mSASSS) (**F**) in patients with AS. * *p* < 0.05

The relationship between the 6 candidate DUSPs and inflammatory markers or disease activity of the individual entities was investigated. As shown in Table 3, an inverse correlation of DUSP22 mRNA levels in T cells with ESR and CRP were observed in patients with AS (*r* = −0.501, *p* < 0.001 and *r* = −0.369, *p* = 0.005, respectively) (Fig. 3A, B). In addition, there was a positive correlation between DUSP7 mRNA levels in T cells and ESR (*r* = 0.310, *p* = 0.038). The mRNA levels of DUSP22 in T cells were negatively associated with BASDAI score (*r* = −0.343, *p* = 0.016, Fig. 3C), but not with BSAFI and BAS-G scores (Fig. 3D, E). In contrast, there was no correlation between the gene expression of other 5 candidate DUSPs in T cells and disease activity scores of AS (all *p* > 0.05) (Fig. S4). Furthermore, there was no significant association between the gene expression of all 6 candidate DUSPs in T cells and mSASSS in this cohort (all *p* > 0.05) (Figs. 3F and S4F).

**Table 3:** Correlations between the mRNA levels of DUSPs in peripheral T cells with disease activity in patients with ankylosing spondylitis

	DUSP4		DUSP5		DUSP6		DUSP7		DUSP14		DUSP22	
	*r*	*p* value	*r*	*p* value	*r*	*p* value	*r*	*p* value	*r*	*p* value	*r*	*p* value
Inflammatory markers												
ESR (mm/h)	0.220	0.121	0.208	0.143	0.221	0.099	0.310	0.038^*^	0.218	0.104	−0.501	< 0.001^*^
CRP (mg/dL)	0.093	0.517	0.072	0.615	0.184	0.171	0.141	0.357	0.101	0.454	−0.369	0.005^*^
Disease activity												
BASDAI	0.145	0.354	0.190	0.223	0.177	0.223	0.156	0.356	0.140	0.338	−0.343	0.016^*^
BASFI	0.099	0.529	0.103	0.510	0.103	0.481	0.074	0.662	−0.010	0.944	−0.231	0.110
BAS-G	−0.138	0.377	−0.068	0.664	0.022	0.882	−0.172	0.308	−0.126	0.390	−0.206	0.156
mSASSS	0.083	0.566	0.032	0.826	−0.046	0.738	0.009	0.954	0.080	0.560	−0.024	0.860

*Abbreviation*: *BASDAI* Bath Ankylosing Spondylitis Disease Activity Index, *BASFI* BAS Functional Index, *BAS-G* BAS Patient Global Score, *CRP* C-reactive protein, *ESR* Erythrocyte sedimentation rate, *h* hour, *mSASSS* the modified Stoke Ankylosing Spondylitis Spinal Score

*Significant *p* value (< 0.05)

**Fig. 4:**
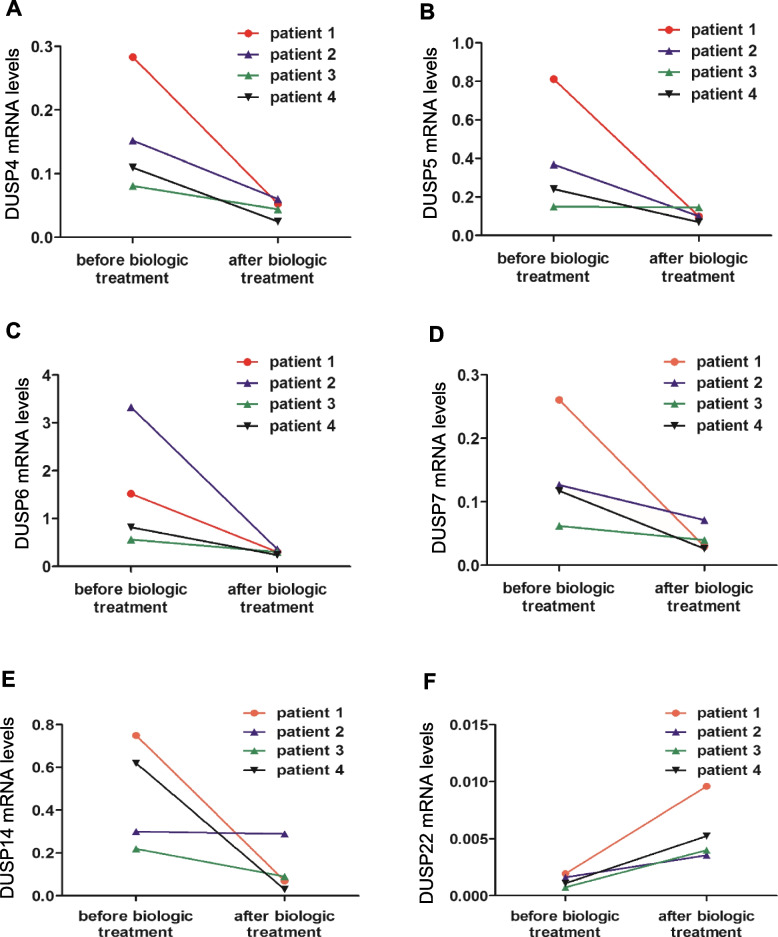
The mRNA levels of DUSPs in peripheral T cells are good biomarkers for treatment response in ankylosing spondylitis. Longitudinal changes in DUSP4 (**A**), DUSP5 (**B**), DUSP6 (**C**), DUSP7 (**D**), DUSP14 (**E**), and DUSP22 (**F**) mRNA levels were determined using peripheral blood T cells of four AS patients after biologic therapy

A follow-up study was performed using peripheral blood T cells from four AS patients with biological treatment. Individual DUSP mRNA levels in peripheral T cells were measured before and after biologic therapy. Disease activity was decreased in all four AS patients. Interestingly, the mRNA levels of DUSP4, DUSP5, DUSP6, DUSP7, and DUSP14 were decreased in T cells of all four AS patients after biologic treatment. In contrast, DUSP22 mRNA levels in T cells of the AS patients were increased after biologic treatment (Fig. 4). These results suggest that expression of the DUSP4, DUSP5, DUSP6, DUSP7, DUSP14, and DUSP22 genes is correlated with the disease activity of AS patients.

### DUSP22 KO mice spontaneously develop AS-like bone disease

**Fig. 5:**
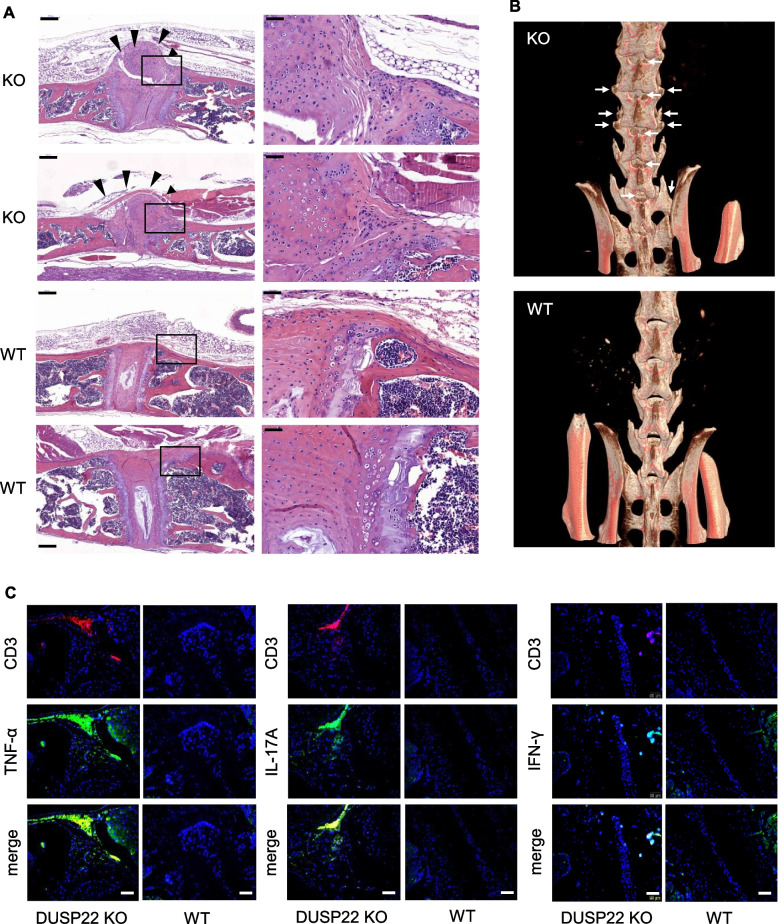
Aged DUSP22 knockout mice spontaneously develop AS-like bone disease. **A** Hematoxylin and eosin-stained sections of vertebral bones from two aged (1-year-old) DUSP22 knockout (KO) mice showed massive mesenchymal cell proliferation and excessive matrix formation bridging across adjacent vertebrae (black arrowheads). Intact joints and intervertebral discs were shown in wild-type (WT) mice. Original magnification, ×10 (left) and × 40 (right). Scale bar, 200 μm (left) and 50 μm (right). **B** Micro-CT radiographs showed the bone structure of spinal joints, sacroiliac joints, and hip joints of DUSP22 KO and wild-type (WT) mice. Arrows denote osteophytes and bony fusion. **C** Immunofluorescence assay showed the increase of TNF-α^+^, IL-17A^+^, and IFN-γ^+^ CD3^+^ T cells in the vertebrae of aged (1-year-old) DUSP22 KO mice. Samples were stained for Alexa Fluor® 488- or PE-conjugated anti-human CD3 antibody, APC-conjugated anti-human TNF-α antibody, FITC-conjugated anti-human IL-17A antibody, and FITC-conjugated anti-human IFN-γ antibody. 4′,6-diamidino-2-phenylindole (DAPI) was used as counterstaining for cell nuclei. Scale bar, 50 μm. One representative image of at least 6 fields is shown

The data derived from T cells of AS patients in our cohort showed that DUSP22 levels were inversely correlated with ESR, CRP, and BASDAI of AS patients. To study whether the reduction of DUSP22 gene expression contributes to AS pathogenesis, we generated a DUSP22 knockout (KO) mouse line using the CRISPR-Cas9 approach (Fig. S5A, B). The expression of DUSP22 was obliterated in peripheral blood T cells and splenic T cells of DUSP22 KO mice (Fig. S5B), and T cell development was normal in DUSP22 KO mice (Fig. S5). Our previous publication demonstrates that the 48-week-old DUSP22 KO mice spontaneously develop inflammation in the lung, liver, and kidney [11]. In this study, we investigated whether aged DUSP22 KO mice spontaneously develop AS-like symptoms. To assess the histologic changes of the spine in DUSP22 KO mice, 1-year-old DUSP22 KO and wild-type mice were sacrificed to examine the morphology of mouse vertebrae. H & E staining showed massively excessive matrix formation resembling a syndesmophyte in DUSP22 KO mice (Fig 5A). Safranin O-fast green staining showed cartilage destruction in the spinal joint of DUSP22 KO mice (Fig. S6A). In contrast, intact joints without excessive matrix formation and normal cartilage structure were shown in the spine of wild-type mice (Fig. 5A and Fig. S6). To detect the spine architecture of mice, the spine of mice was scanned by micro-computed tomography (μCT). The μCT images showed osteophytes and bony fusion in the spinal joints and sacroiliac joints of DUSP22 KO mice (Fig. 5B). The images also showed joint space narrowing in DUSP22 KO mice (Fig. 5B). Collectively, these results suggest that aged (1-year-old) DUSP22 KO mice spontaneously develop AS-like bone disease.

### Higher expression of TNF-α^+^, IL-17A^+^, and IFN-γ^+^ T cells in the vertebrae of aged DUSP22 KO mice

Our previous publication reported that DUSP22 KO T cells produce more IFN-γ and IL-17A upon antigen re-stimulation [11]. Moreover, 24-week-old DUSP22 KO mice display increased serum levels of the proinflammatory cytokines IFN-γ, IL-17A, and TNF-α [11]. To evaluate the inflammatory cytokine levels in the vertebrae of DUSP22 KO mice, we performed immunofluorescence imaging using the spine sections of DUSP22 KO and wild-type mice. The imaging data showed that the population of CD3^+^ T cells was increased in the vertebrae of the aged DUSP22 KO mice compared to that of wild-type mice (Fig. 5C and Fig. S7). Interestingly, TNF-α^+^, IL-17A^+^, and IFN-γ^+^ T cells were also markedly increased (Fig. 5C and Fig. S7). TNF-α and IFN-γ are osteoblastogenic factors; however, the function of IL-17A on osteoblastogenesis is still controversial [33]. To evaluate the osteoblast activity in the spine of symptomatic mice, we performed immunohistochemistry analyses using anti-osteocalcin antibody. Osteocalcin levels were increased in the spinal joint of DUSP22 KO mice (Fig. S6B), suggesting an increased activity of osteoblasts in the spine of DUSP22 KO mice. These results suggest that the osteophytes and bony fusion in the spine of DUSP22 KO mice may be due to the cytokine-induced osteoblast activation.

## Discussion

The pathogenesis of AS remains largely unknown, and early diagnostic biomarkers for disease activity of AS are lacking. Several DUSPs play important roles in inflammatory diseases through mediating T cell-mediated immune responses and regulating proinflammatory cytokine production [4, 5, 11, 25, 34, 35]. In the current study, we found that six DUSP-family phosphatases in T cells may be useful for better diagnosis of AS. Also, there was a significant association between the gene expression levels of several DUSPs and inflammatory cytokines in T cells. Furthermore, several DUSPs (particularly DUSP22) were identified to be potential biomarkers of disease activity for AS. Finally, aged DUSP22 knockout mice spontaneously developed the AS-like phonotypes with the induction of inflammatory cytokines. These findings provide strong evidence for the notable role of DUSPs, particularly DUSP22, in the pathogenesis of AS.

The diagnosis of AS is still based upon 1984 modified New York criteria [27], which include positive radiologic findings and clinical inflammatory back pain. However, the negative HLA- B27, lack of family history, and the absence of typical inflammatory back pain and radiographic sacroiliitis in the early stage may lead to delayed diagnosis of AS [36–38]. The diagnosis of AS is frequently delayed by 6 to 10 years, resulting in a poor disease outcome [37, 39]. Therefore, there is an unmet need of early diagnostic biomarkers for AS [40]. We reported that DUSPs regulate T cell-mediated immunity [1, 11, 12, 25]. In this study, mRNA levels of DUSP4, DUSP5, DUSP6, DUSP7, and DUSP14 in peripheral T cells were significantly higher in AS group than HCs, while DUSP22 mRNA levels were significantly lower in AS group than HCs. Among them, DUSP22 showed the greatest potential to detect AS (AUC > 0.9). Because of small sample sizes, further studies are needed to confirm whether DUSP22 indeed can be used as a diagnostic biomarker for AS.

Inflammatory cytokines play critical roles in the pathogenesis of AS. HLA-B27 is responsible for SpA by inducing secretion of proinflammatory cytokines [15]. DUSP22 mRNA level in AS T cells was found to be negatively correlated with TNF-α mRNA levels. In contrast, the mRNA levels of DUSP4, DUSP5, DUSP6, DUSP7, and DUSP14 in AS T cells were positively correlated with TNF-α mRNA levels. These results suggested that DUSP22 may play a distinct role in the regulation of T cell cytokines when compared to other DUSPs. TNF-α plays a central role in AS, and the treatment with TNF-α blocker improves inflammatory signs and symptoms of AS [22]. We previously reported that TNF-α and IL-1β could enhance bone morphogenetic protein (BMP) 2 expression in PBMCs from AS patients, and the increase of BMP2 is associated with radiographic progression [41]. Taken together, these data imply that several DUSP-family phosphatases are associated with proinflammatory cytokine expression and are involved in pathogenesis of AS through regulation of T cell-mediated inflammation.

We analyzed the relationship between mRNA expression of DUSPs in T cells and the levels of serum inflammatory markers and found that DUSP22 showed high statistical significance; an inverse correlation of DUSP22 to serum ESR and CRP levels was found in our AS patients. Moreover, DUSP22 gene expression in T cells was negatively correlated with BASDAI, which was a measure of main symptoms experienced by AS patients. Similarly, DUSP22 level in synovium or serum was negatively correlated with CRP level and disease activity score in 28 joints (DAS28-ESR) in patients with RA [26]. In this report, the longitudinal observations suggest that DUSP22 levels can serve as a good biomarker for treatment response in AS patients. Furthermore, DUSP22 KO mice developed AS-like bone disease. Taken together, the reduction of DUSP22 levels may reflect the inflammatory status and disease activity of AS and could be used as a biomarker for disease activity and treatment response.

Advanced AS was characterized by a fusion of the vertebral bodies due to bridging syndesmophytes. Notably, spontaneous syndesmophyte formation was observed in aged DUSP22 KO mice in the current study. In addition, TNF-α^+^, IL-17A^+^, and IFN-γ^+^ CD3^+^ T cells were markedly increased in vertebrae of aged DUSP22 KO mice. We reported that elevated serum levels of the proinflammatory cytokines TNF-α, IL-17A, and IFN-γ in DUSP22 KO mice [11]. Importantly, TNF-α and IL-17A are involved in the development of AS and the formation of syndesmophytes [17, 21, 41–43]. Similar to TNF-α, serum IFN-γ levels are also increased in AS patients [44]; the induction of serum IFN-γ or TNF-α is correlated with the disease activity of AS patients [44]. These data implied that DUSP22 may play a crucial role in the development of AS through regulation of Th1 and Th17 cytokine secretion. It is likely that DUSP22 deficiency in other type cells, such as chondrocytes or osteoclasts, osteoblasts, also contribute to the development of AS-like phenotypes in the DUSP22 whole-body knockout mouse line. Similar to DUSP22 knockout mice, secretion of Th1 and Th17 cytokines is also induced in another AS-like mouse model, T cell-specific SHP2 conditional knockout (Ptpn11^f/f^;CD4-Cre) mice [45]. Furthermore, SHP2 deficiency in chondrocytes also contributes to development of AS-like phenotypes in 1-year-old Ptpn11^f/f^;CD4-Cre mice [45]. Both AS-like mouse models in this study and the previous publications [45, 46] suggest that induction of T cell-medicated inflammation in combination with activation of chondrocytes, osteoblasts, or other cell types play critical roles in the development of AS phenotypes. These findings also suggest that the influence of phosphatases on the pathogenesis of AS may be universal.

The limitation of this study is the relatively small number of enrolled subjects. Further studies with a larger sample size are needed to confirm our findings. In addition, there was no correlation between DUSP22 gene expression in T cells and mSASSS in our patients with AS. The natural course of radiographic progression in AS is slow; the mean change of mSASSS is 1.3 per year [47]. Therefore, long-term longitudinal follow-up study is required to investigate the influence of DUSP22 on the radiographic change of AS. Besides DUSP22, five other DUSPs (DUSP4, DUSP5, DUSP6, DUSP7, DUSP7, and DUSP14) may be useful for the diagnosis of AS; however, a longitudinal study with a larger sample size is also necessary to evaluate whether the expression levels of these five DUSPs can guide the treatment AS. Besides immune factors, it would be valuable to further study the effect of DUSPs, including DUSP22, on pathological osteogenesis. Another limitation of this study is that the correlation of gene expression of DUSPs and IL-17A was not evaluated because IL-17A mRNA level in peripheral blood T cells was extremely low and undetectable. Thus, it is unclear whether DUSPs play a role in IL-17A-mediated T cell inflammatory response in AS patients. Furthermore, because HLA-B27 is strongly correlated with the development of AS, the roles of DUSPs in HLA-B27 regulation need to be studied in the future.

## Conclusions

**Fig. 6:**
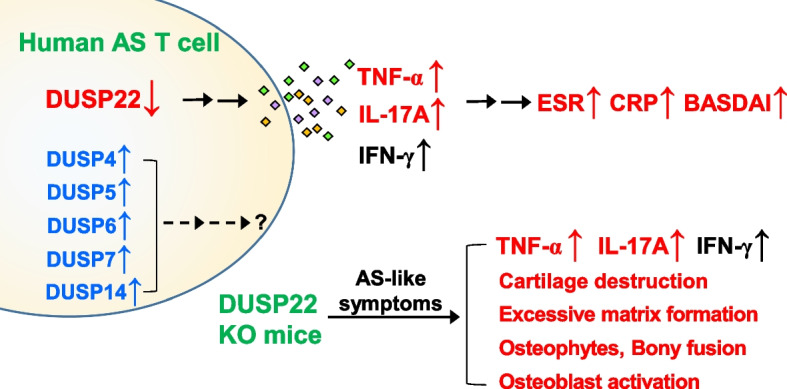
Schematic diagram of the correlation of DUSPs and ankylosing spondylitis. The mRNA levels of DUSP4, DUSP5, DUSP6, DUSP7, and DUSP14 were increased in peripheral blood T cells from ankylosing spondylitis (AS) patients, whereas the mRNA levels of DUSP22 were decreased. Downregulation of the DUSP22 protein in T cells of AS patients was concomitant to the induction of the proinflammatory cytokines TNF-α, IL-17A, and IFN-γ. DUSP22 downregulation was correlated with the values/levels of ESR, CRP, BASDAI, TNF-α, IL-17A, and IFN-γ of AS patients. Consistently, DUSP22 knockout (KO) mice spontaneously developed AS-like symptoms with induction of TNF-α, IL-17A, and IFN-γ. It is unclear whether IFN-γ contributes to the pathogenesis of AS. In addition, the mRNA levels of DUSP4, DUSP5, DUSP6, DUSP7, and DUSP14 were modestly correlated with TNF-α mRNA levels in the T cells of AS patients. The roles of DUSP4, DUSP5, DUSP6, DUSP7, and DUSP14 in the pathogenesis of AS are unclear. These findings suggest that DUSP22 downregulation in T cells plays a critical role in the pathogenesis of AS. ESR, erythrocyte sedimentation rate; CRP, C-reactive protein; BASDAI, Bath Ankylosing Spondylitis Disease Activity Index

In summary, this is the first study to investigate the role of DUSP-family phosphatases in patients with AS. We found that six DUSPs may be involved in T cell-related cytokine production and play crucial roles in the regulation of disease activity of AS (Fig. 6). In particular, diminished DUSP22 in T cells is associated with increased T cell-mediated inflammation, resulting in AS development (Fig. 6).

## Supplementary Information

**Additional file 1: Fig. S1.** Purity of the isolated human T cells is more than 90%. **Fig. S2.** Correlations between the mRNA levels of DUSPs and proinflammatory cytokines in peripheral T cells in patients with ankylosing spondylitis. **Fig. S3.** Increased proinflammatory cytokines and decreased DUSP22 protein levels are shown in T cells of AS patients **Fig. S4.** Correlations between the mRNA levels of DUSPs in peripheral T cells and disease activity in patients with ankylosing spondylitis. **Fig. S5.** DUSP22 knockout mice display normal T cell development. **Fig. S6.** DUSP22 KO mice display cartilage destruction and osteocalcin induction in the spinal joint. **Fig. S7.** Aged DUSP22 KO mice display the increase of inflammatory T cells in the vertebrae.

**Additional file 2: Table S1.** Probes and primers of Q-PCR for human DUSP mRNA levels. **Table S2.** Demographic characteristics of the second batch of AS patients and healthy controls.

**Additional file 3.** Images of the original blots. The uncropped blots of Fig. S5A.

## Data Availability

Data and materials are available upon reasonable request.

## Abbreviations

AS: Ankylosing spondylitis
AUC: Area under the curve
BASDAI: Bath Ankylosing Spondylitis Disease Activity Index
BASFI: Bath Ankylosing Spondylitis Functional Index
BAS-G: Bath Ankylosing Spondylitis Patient Global Score
BMP: Bone morphogenetic protein
CI: Confidence interval
CRP: C-reactive protein
DAPI: Diamidino-2-phenylindole
DUSP: Dual-specificity phosphatases
EDTA: Ethylenediaminetetraacetic acid
ESR: Erythrocyte sedimentation rate
FAK: Focal adhesion kinase
H&E: Hematoxylin and eosin
HLA: Human leukocyte antigen
IFN-γ: Interferon-γ
IL: Interleukin
JKAP: JNK pathway-associated phosphatase
KO: Knockout
mSASSS: Modified Stokes Ankylosing Spondylitis Spinal Score
PBMC: Peripheral blood mononuclear cell
qRT-PCR: Quantitative real-time polymerase chain reaction
ROC: Receiver operator characteristics
SpA: Seronegative spondyloarthropathies
STAT: Signal transducer and activator of transcription
Th: T helper
Th1: T helper type 1
TNF-α: Tumor necrosis factor-α
