# Supplementary material for: Dual-specificity phosphatases 22-deficient T cells contribute to the pathogenesis of ankylosing spondylitis
Source: BMC Med. 2023 Feb 10;21:46. doi: 10.1186/s12916-023-02745-6 (PMC9921195; doi:10.1186/s12916-023-02745-6)
Supplement: Supplementary file 1 — Additional file 1: Fig. S1. Purity of the isolated human T cells is more than 90%. Fig. S2. Correlations between the mRNA levels of DUSPs and proinflammatory cytokines in peripheral T cells in patients with ankylosing spondylitis. Fig. S3. Increased proinflammatory cytokines and decreased DUSP22 protein levels are shown in T cells of AS patients Fig. S4. Correlations between the mRNA levels of DUSPs in peripheral T cells and disease activity in patients with ankylosing spondylitis. Fig. S5. DUSP22 knockout mice display normal T cell development. Fig. S6. DUSP22 KO mice display cartilage destruction and osteocalcin induction in the spinal joint. Fig. S7. Aged DUSP22 KO mice display the increase of inflammatory T cells in the vertebrae. [file 12916_2023_2745_MOESM1_ESM.pdf]

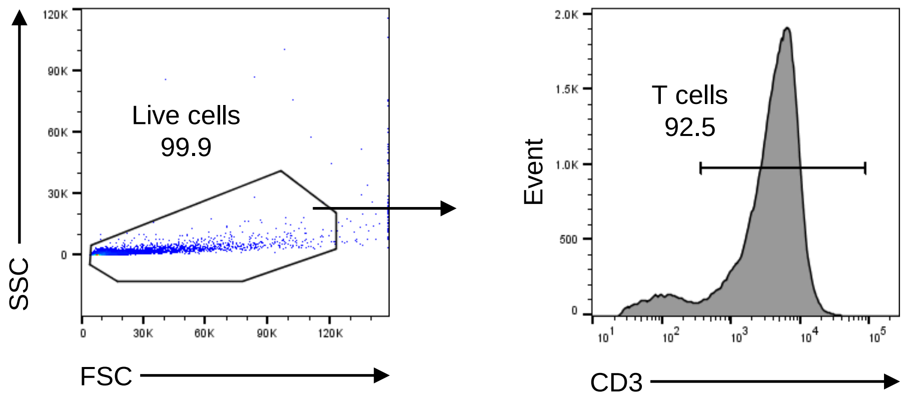

Fig. S1. Purity of the isolated human T cells is more than 90%. Flow cytometry analyses of CD3-positive T cells in the negatively isolated T cells from peripheral blood leukocytes of a representative AS patient.

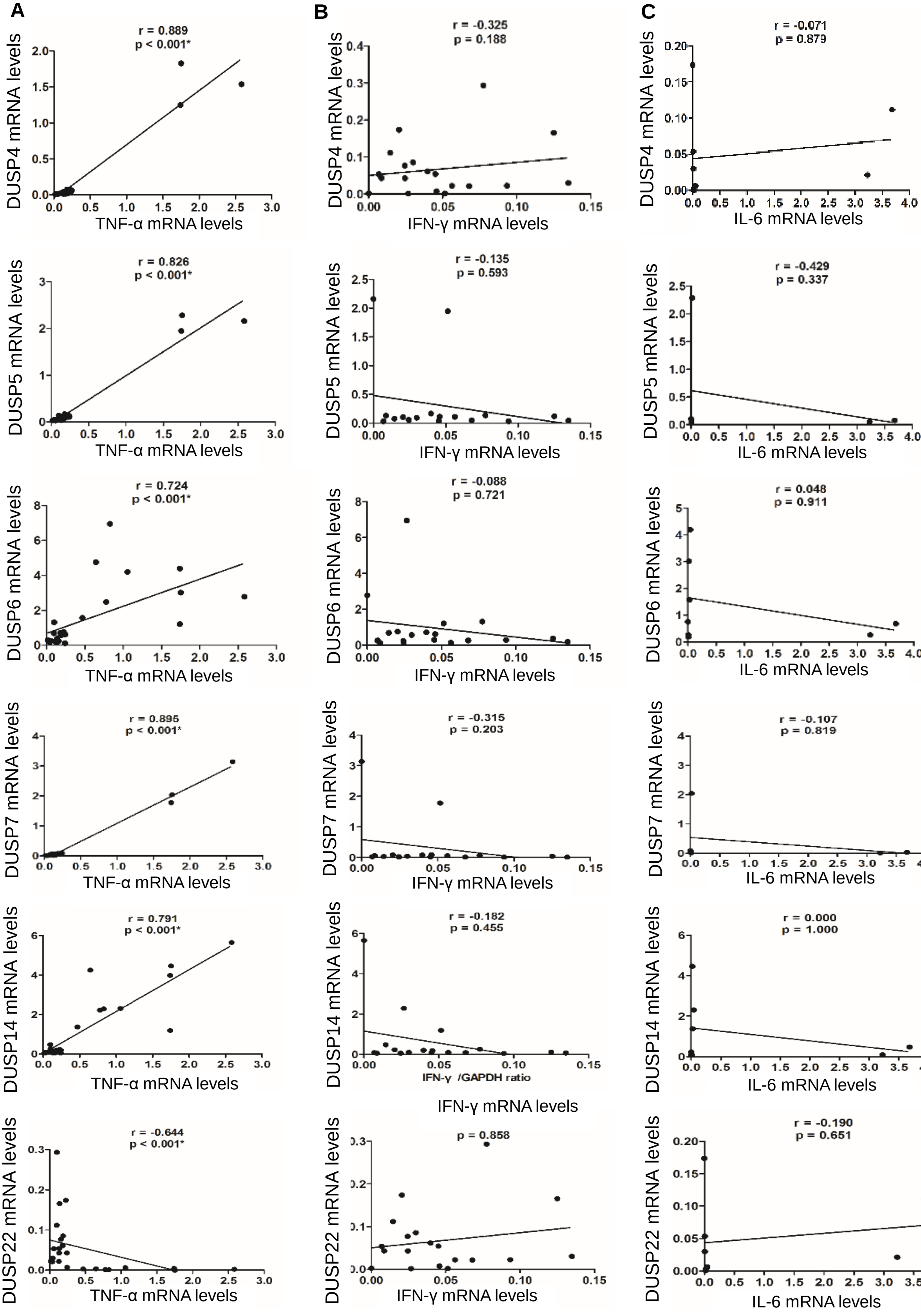
A
B
C
r = 0.889
p < 0.001*
DUSP4 mRNA levels
TNF-α mRNA levels
r = -0.325
p = 0.188
DUSP4 mRNA levels
IFN-γ mRNA levels
r = -0.071
p = 0.879
DUSP4 mRNA levels
IL-6 mRNA levels
r = 0.826
p < 0.001*
DUSP5 mRNA levels
TNF-α mRNA levels
r = -0.135
p = 0.593
DUSP5 mRNA levels
IFN-γ mRNA levels
r = -0.429
p = 0.337
DUSP5 mRNA levels
IL-6 mRNA levels
r = 0.724
p < 0.001*
DUSP6 mRNA levels
TNF-α mRNA levels
r = -0.088
p = 0.721
DUSP6 mRNA levels
IFN-γ mRNA levels
r = 0.048
p = 0.911
DUSP6 mRNA levels
IL-6 mRNA levels
r = 0.895
p < 0.001*
DUSP7 mRNA levels
TNF-α mRNA levels
r = -0.315
p = 0.203
DUSP7 mRNA levels
IFN-γ mRNA levels
r = -0.107
p = 0.819
DUSP7 mRNA levels
IL-6 mRNA levels
r = 0.791
p < 0.001*
DUSP14 mRNA levels
TNF-α mRNA levels
r = -0.182
p = 0.455
DUSP14 mRNA levels
IFN-γ /GAPDH ratio
IFN-γ mRNA levels
r = 0.000
p = 1.000
DUSP14 mRNA levels
IL-6 mRNA levels
r = -0.644
p < 0.001*
DUSP22 mRNA levels
TNF-α mRNA levels
p = 0.858
DUSP22 mRNA levels
IFN-γ mRNA levels
r = -0.190
p = 0.651
DUSP22 mRNA levels
IL-6 mRNA levels

Fig. S2

Fig. S2. Correlations between the mRNA levels of DUSPs and proinflammatory cytokines in peripheral T cells in patients with ankylosing spondylitis. Association between DUSPs (DUSP4, DUSP5, DUSP6, DUSP7, DUSP14, DUSP22) and TNF-α (A), IFN-γ (B), IL-6 (C) mRNA levels in peripheral blood T cells in patients with ankylosing spondylitis. * $p < 0.05$.

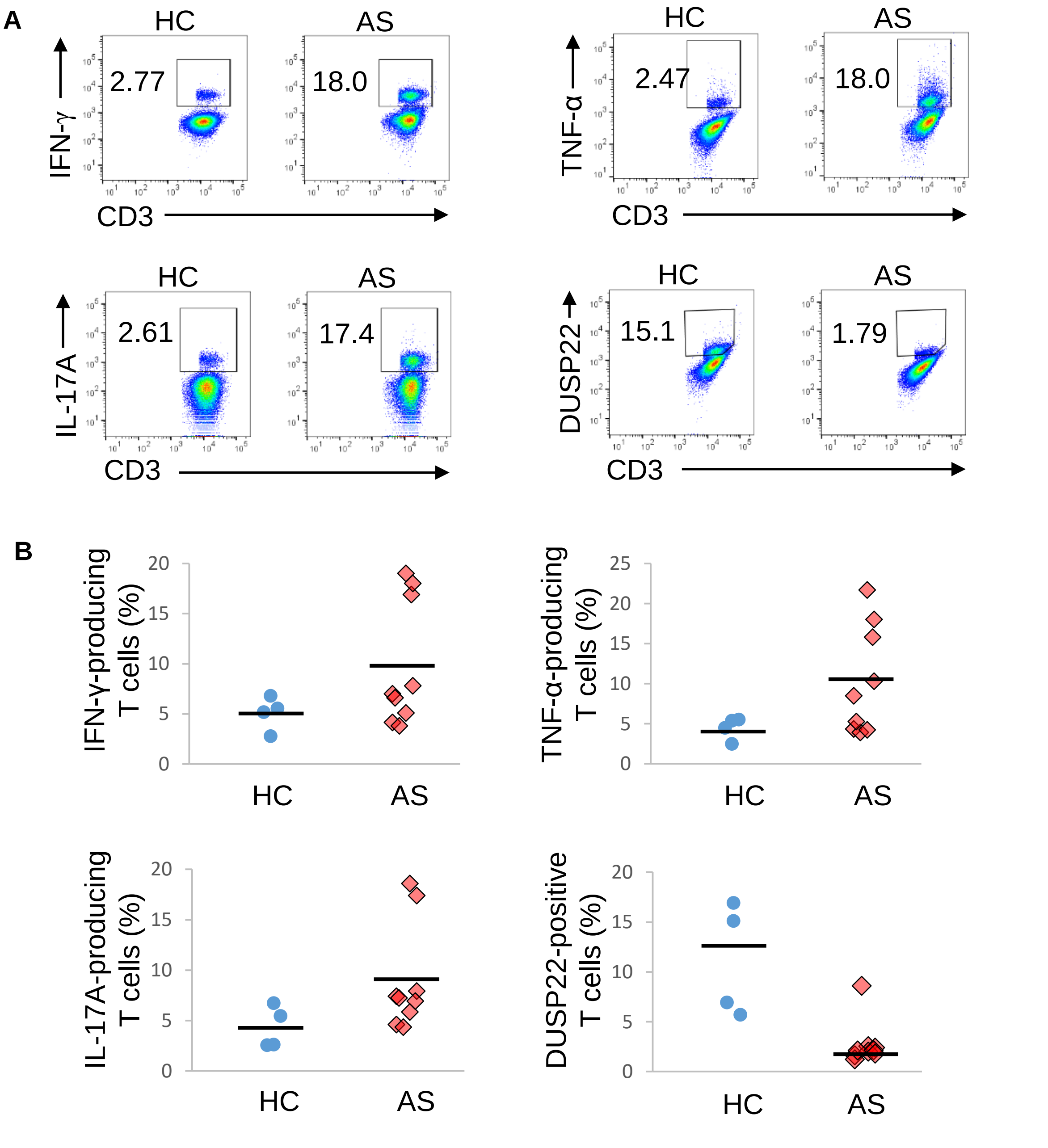

Fig. S3. Increased proinflammatory cytokines and decreased DUSP22 protein levels are shown in T cells of AS patients. (**A**) Flow cytometry analyses of IFN-γ^+, TNF-α^+, IL-17A$^+$, and DUSP22$^+$ T cells [CD3-gated] from peripheral blood leukocytes of 9 AS patients and 4 healthy controls. The frequencies of IFN-γ-, TNF-α-, and IL-17A-producing T cells were increased in AS patients compared to healthy controls, while the frequencies of DUSP22-positive T cells were decreased in AS patients. The results from a representative healthy control (HC) and a representative AS patients are shown (A). (B) Statistical analyses of cytokine-producing T cells or DUSP22-positive T cells in the HC or AS group are shown. Bar, mean of the group values.

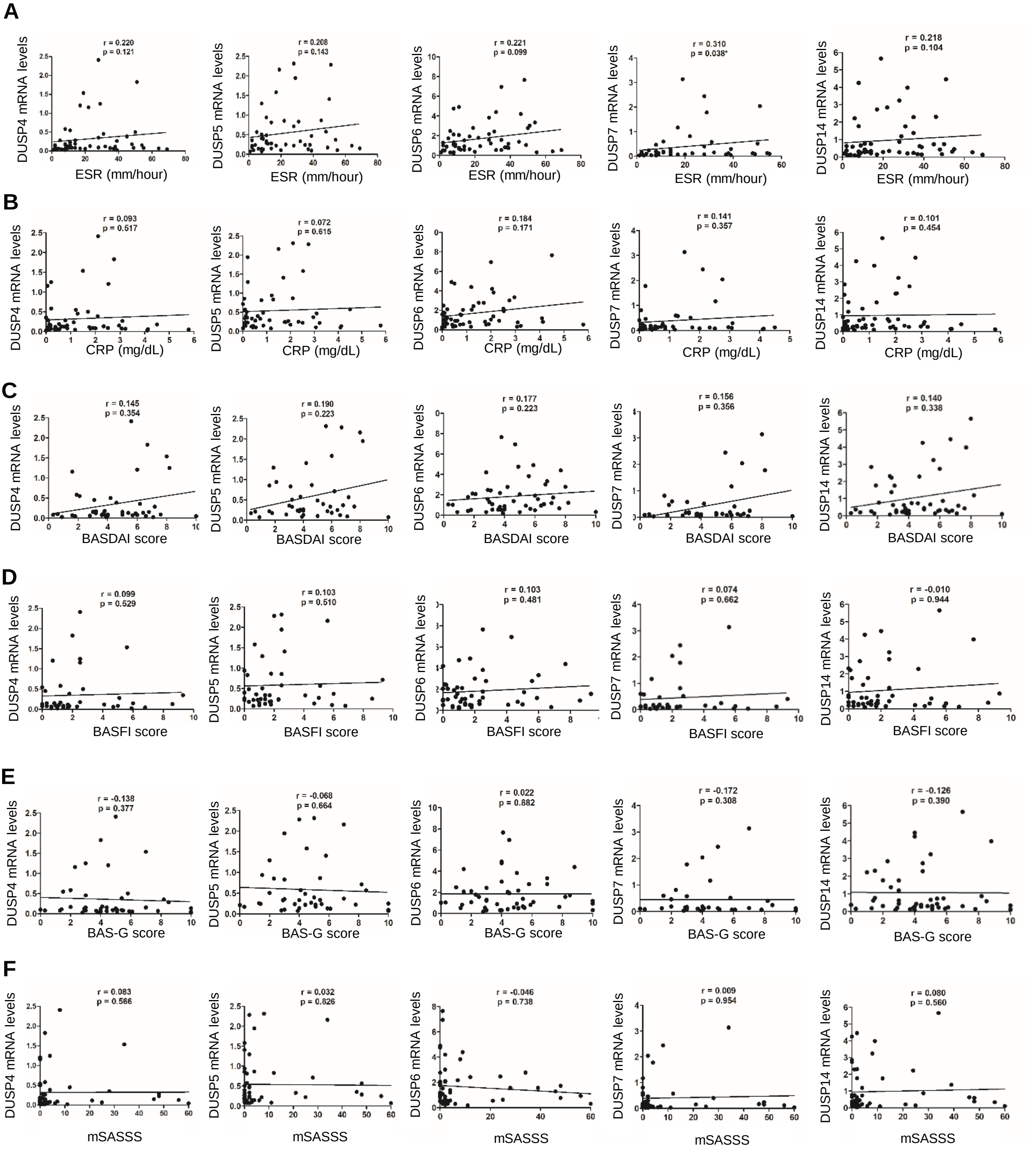

Fig. S4. Correlations between the mRNA levels of DUSPs in peripheral T cells and disease activity in patients with ankylosing spondylitis. Association between DUSPs mRNA levels in peripheral blood T cells and erythrocyte sedimentation rate (ESR) (A), C-reactive protein (CRP) levels (B), Bath Ankylosing Spondylitis Disease Activity Index (BASDAI) (C), BAS Functional Index (BASFI) (D), BAS Patient Global Score (BAS-G) (E), and the modified Stoke Ankylosing Spondylitis Spinal Score (mSASSS) (F) in patients with ankylosing spondylitis. * $p < 0.05$.

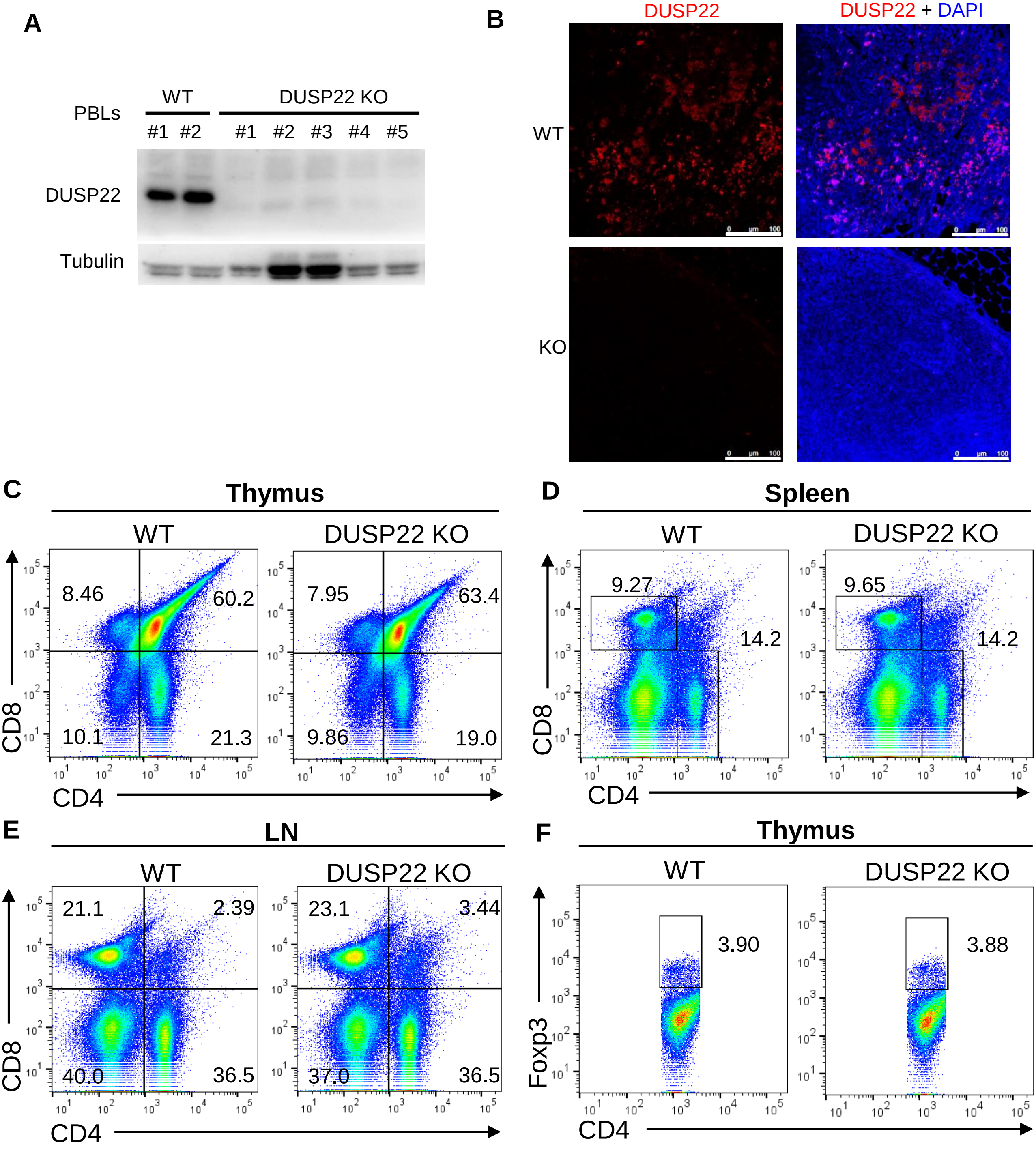

Fig. S5. DUSP22 knockout mice display normal T-cell development. (**A**) Immunoblotting analyses of DUSP22 expression in peripheral blood leukocytes (PBLs) of wild-type (WT) or DUSP22 KO mice. (**B**) Immunofluorescence analyses of DUSP22 expression in the lymph node of WT or DUSP22 KO mice. Scale bar, 100 µm. (**C-F**) Flow cytometry analyses of T cells (C-E) and Treg cells (F) from the thymus, spleen, or lymph nodes of 5-week-old DUSP22 KO or WT mice. Data shown are representative of three independent experiments.

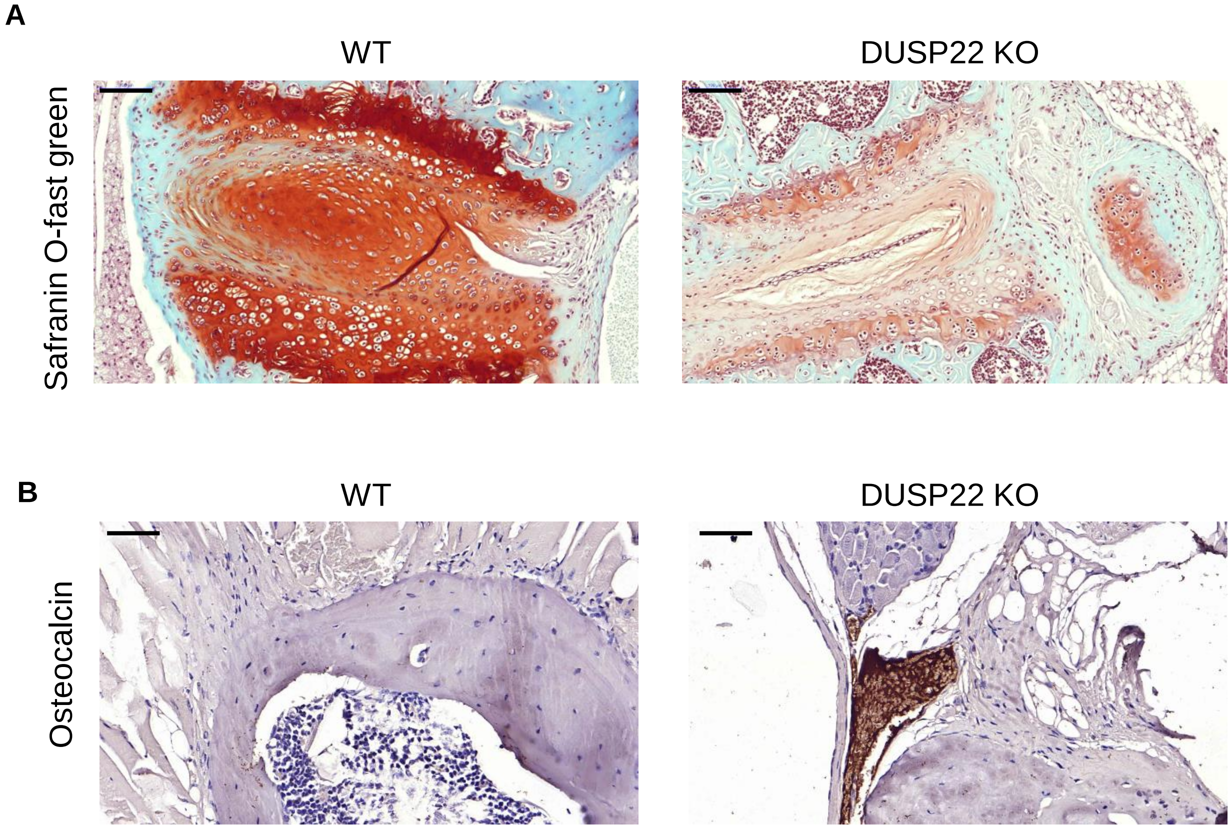

Fig. S6. DUSP22 KO mice display cartilage destruction and osteocalcin induction in the spinal joint. (**A** and **B**) The paraffin-embedded spine section of DUSP22 knockout (KO) or wild-type (WT) mice were subjected to safranin O-fast green staining (A) and osteocalcin staining (B). Scale bar, 100 µm.

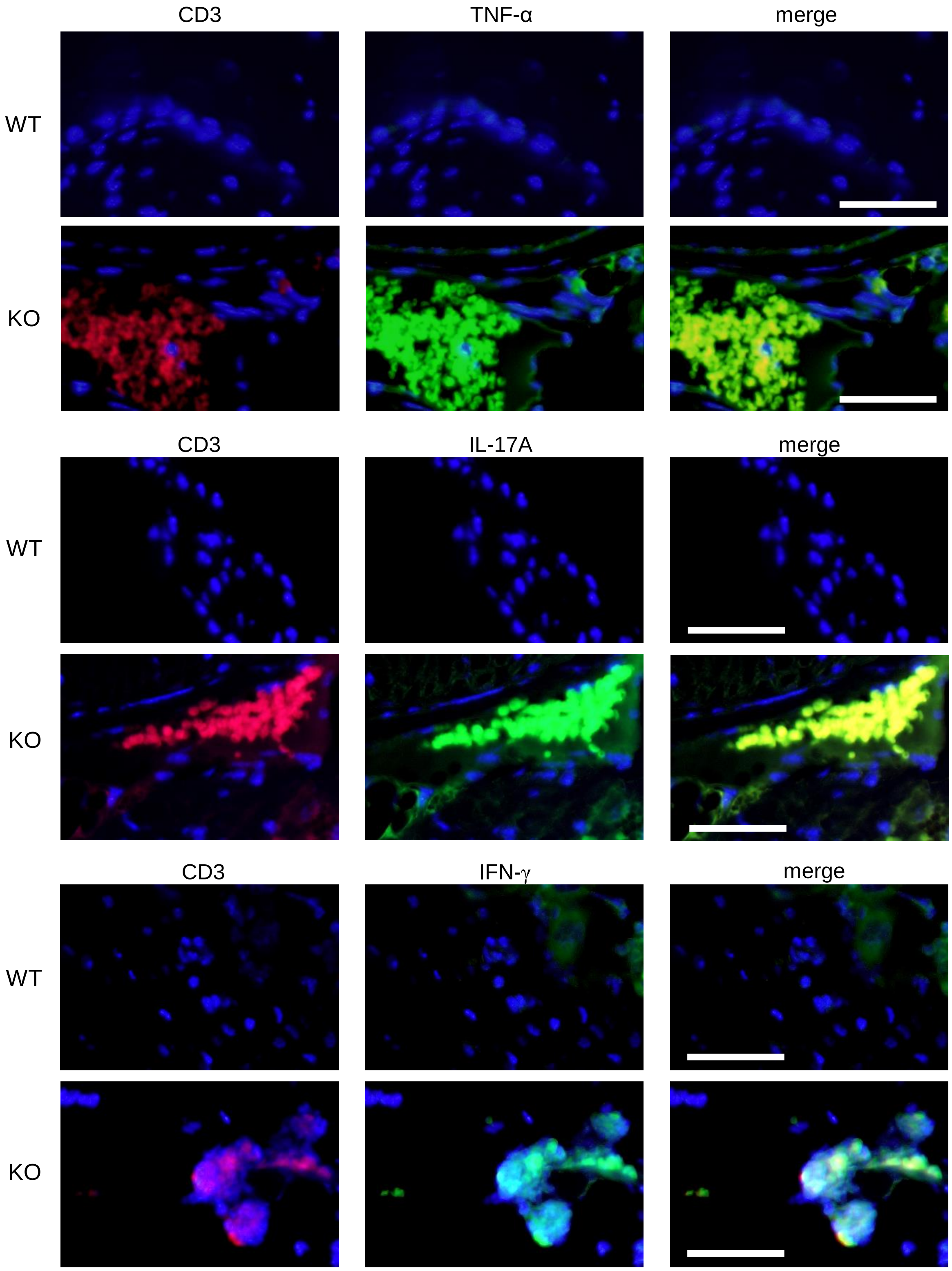

Fig. S7. Aged DUSP22 KO mice display the increase of inflammatory T cells in the vertebrae. High magnification images of Fig. 5 showed the increase of TNF-α$^+$, IL-17A$^+$, and IFN-γ$^+$ CD3$^+$ T cells in the vertebrae of aged DUSP22 KO mice. Scale bar, 50 μm.
