# Supplementary material for: Dual-specificity phosphatases 22-deficient T cells contribute to the pathogenesis of ankylosing spondylitis
Source: BMC Med. 2023 Feb 10;21:46. doi: 10.1186/s12916-023-02745-6 (PMC9921195; doi:10.1186/s12916-023-02745-6)
Supplement: Supplementary file 2 — Additional file 2: Table S1. Probes and primers of Q-PCR for human DUSP mRNA levels. Table S2. Demographic characteristics of the second batch of AS patients and healthy controls. [file 12916_2023_2745_MOESM2_ESM.pdf]

Table S1. Probes and primers of Q-PCR for human DUSP mRNA levels

Gene	Accession Number	Probe Sequence	Sense Primer Sequence	Anti-sense Primer Sequence
DUSP1	NM_004417	AACTCAGCACATTCGGGACCAAT	GCAGTTATGGTGATGACTTA	CGGGAATAATACTGGTAGG
DUSP2	NM_004418	TGAAAGGCTCACAGACAGACACAC	GATGGGTGTGTCATGTTG	CTGAGCACAAATAATTTTCCA
DUSP3	NM_004090	CCCAGTTGTCCTGTTTCTGTAACTT	GCACATAAAGAAGCTTGC	GCATCTTAGGCCTTACAC
DUSP4	NM_001394	TGTATTCGGAGTCCTTATTGCCATTCT	CTCCTTCCCCATCATTTC	GCTCTAAGTTGGAGTGTTC
DUSP5	NM_004419	AGAGCCCTCATCAGCCAGTG	GGATGTAAAACCCATTTCAC	AGCTGACATTTACCACTG
DUSP6	NM_001946	TTCCGCACTTGGTAACCTTGTCTA	TCTGCAATCTACGTGAAA	GGAGCAAATCTCTCTGTTA
DUSP7	NM_001947	TCGCCTGTGATGACTGCTGC	CAGTCTGACTTAGCTCTG	GGCAGAAAATGCAAAGAC
DUSP8	NM_004420	AGTCCTGTTCCCGTTCCCTCT	CTGTCATCCCTCCTTTCA	AACGCAACAGACATATGG
DUSP9	NM_001395	CCCAGTGATCCCGTCAAGACAT	GTCACCTCCCTGTTTCTC	CACCCTCACTCACAAAGA
DUSP10	NM_007207	AGCCACTGACAGCAACAAGCA	GGCCTGTTCAACTACAAG	GCTTCCTCAATGAACTCA
DUSP11	NM_003584	CAACCAGTCCACAATAAGCCTGTTAA	CCAGGTCAAGTGATTTTG	GATGGTGTCTCTGGTAAA
DUSP12	NM_007240	TGTGCTGCCTTTGCTTCTTATCATTC	GGAAGAAACTTGCAGATG	CCATGTAAAGCATAGTTTCC
DUSP13	NM_001007271	CCTAGAGCCTGCTACTCCATCTG	CACAGAAGATGGTGGAAG	CACACTTGATGCTGTTTC
DUSP14	NM_007026	TTAGTGCCACTGAAGCCTGC	ACACCTGATGCCTTACTG	CCTCCCTCCCTATCTCTTA
DUSP15	NM_080611	ATCCACTGCTGCCGCCTTAAT	GCACTTCAAAGAATGTATCA	CGTCACAGTCATCACATA
DUSP16	NM_030640	TTCCCGTGACCTGTTCTCTGAC	GAGAGCCCCTTTGAAAAG	CCGAAAAGCTAGACTGAC
DUSP18	NM_152511	ACCCTGGCTCAACCTGTTACCCG	CGTTGCCGTAGAGATGG	GGTCTTCAGAGAAATCCTGG
DUSP19	NM_080876	CTCTTGATATGTACGAAGCTGCTCCAT	TCCTGATGAATTCTGAACAA	CCTGTATTCTGTCACACTTA
DUSP21	NM_022076	CCCTTTAACAAGGCACCATCCG	GCACCAAGACTGAACTTG	CGTTCAAAACTCCAACCC
DUSP22	NM_020185	AATATGTGTCACCTTGTTCTTGCTCA	CCCTGTAACATGCCATAG	GCACTATCATGGACAGAC
DUSP23	NM_017823	AGCAGTCTTCCAGTTCTACCAGC	GAGACCTATGAGCAGGAG	CAGTGTCTTCAGCCAATG
DUSP24	NM_016086	CATCACCTTGGCTCTGTCATTCTG	GTGTCACTTCATTGAAATTCA	TGAGGTAGGCTATGATGG
DUSP26	NM_024025	TCTCTTCGCTGTCTGTCTCTATGCT	CTCTCTCCCTCACCAAAA	GGGTTCTCTGTCTGGTTC
DUSP27	NM_001080426	AGACCTCATCCACCTCATTCCAG	TCAGGGAGAAGATGGATG	CACACTCTTCTCAGCTATG
GAPDH	NM_002046	CCTTCATTGACCTCAACTACATGGTT	CTGGTAAAGTGGATATTGTTG	CCCCACTTGATTTTGGAG

Table S2. Demographic characteristics of the second batch of AS patients and healthy controls

	AS<br>N = 9	Healthy controls<br>N = 4	*p* value
Age, years	49.8 ± 12.4	41.5 ± 2.1	0.148
Gender, male	4 (44.4)	2 (50.0)	1.000
Disease duration, years	17.0 (10.5, 25.5)	-	-
HLA-B27 positivity	9 (100.0)	-	-
mSASSS (0-72)	2 (0, 19)	-	-
Sacroiliitis grade (0-4)	2 (2, 3.5)	-	-
ESR, mm/hour	13.6 ± 9.0	-	-
CRP, mg/dl	0.1 (0.1, 0.4)	-	-
BASDAI (0-10)	2.0 ± 0.8	-	-
BASFI score (0-10)	2.9 ± 1.9	-	-
BAS-G score (0-10)	2.4 ± 1.9	-	-
Biologic used	0 (0.0%)	-	-

Abbreviation: BASDAI, Bath Ankylosing Spondylitis Disease Activity Index; BASFI, BAS Functional Index; BAS-G, BAS Patient Global Score; CRP, C-reactive protein; ESR, erythrocyte sedimentation rate; HLA, human leukocyte antigen; mSASSS, modified Stokes Ankylosing Spondylitis Spinal Score.
Data are presented as frequency (percentage), mean ± standard deviation, or median (interquartile range).
