# Supplementary figures and images for: Dual-specificity phosphatases 22-deficient T cells contribute to the pathogenesis of ankylosing spondylitis
Source: BMC Med. 2023 Feb 10;21:46. doi: 10.1186/s12916-023-02745-6 (PMC9921195; doi:10.1186/s12916-023-02745-6)

**Fig. S5A**

**DUSP22**

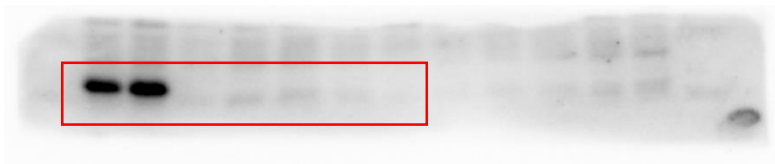

**Tubulin**

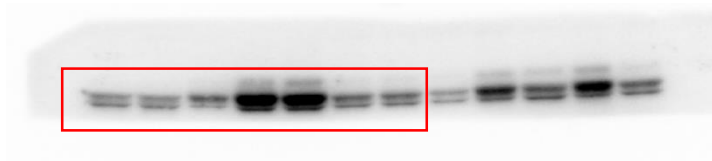

Supplement: Supplementary file 3 — Additional file 3. Images of the original blots. The uncropped blots of Fig. S5A. [file 12916_2023_2745_MOESM3_ESM.pdf]
